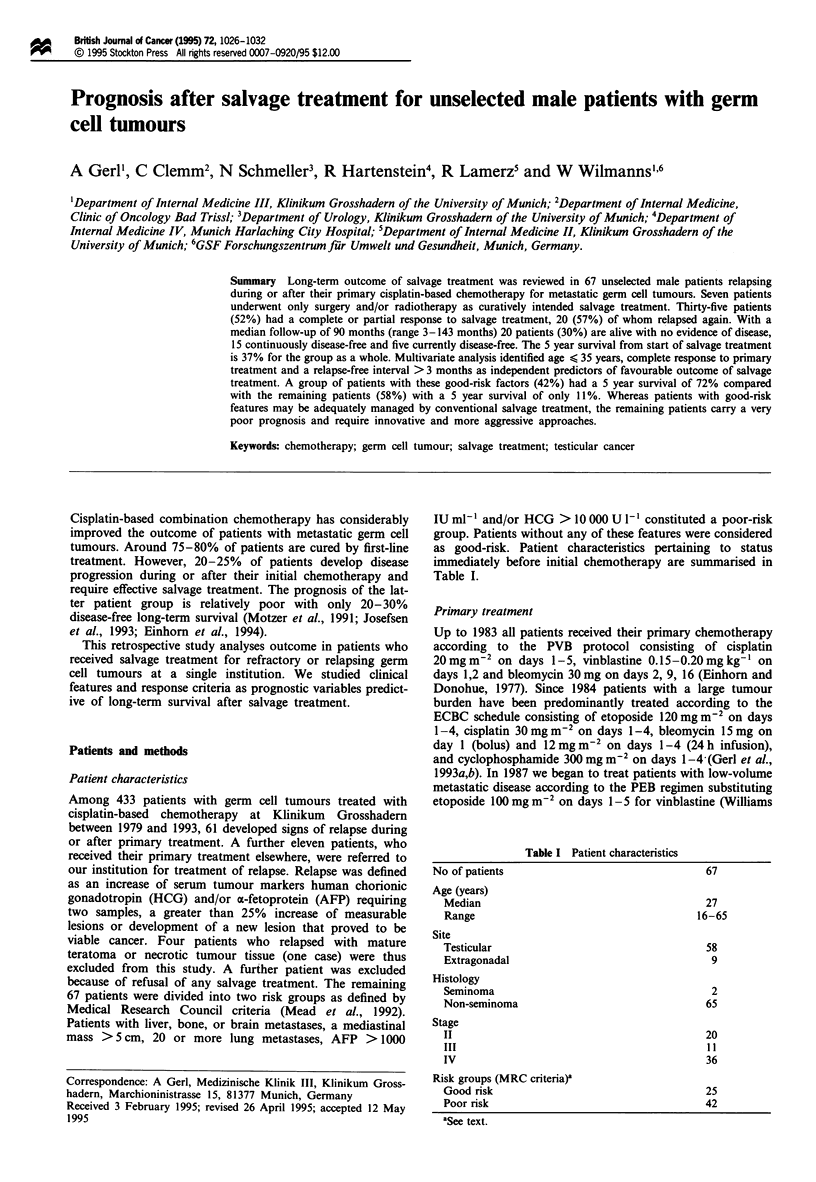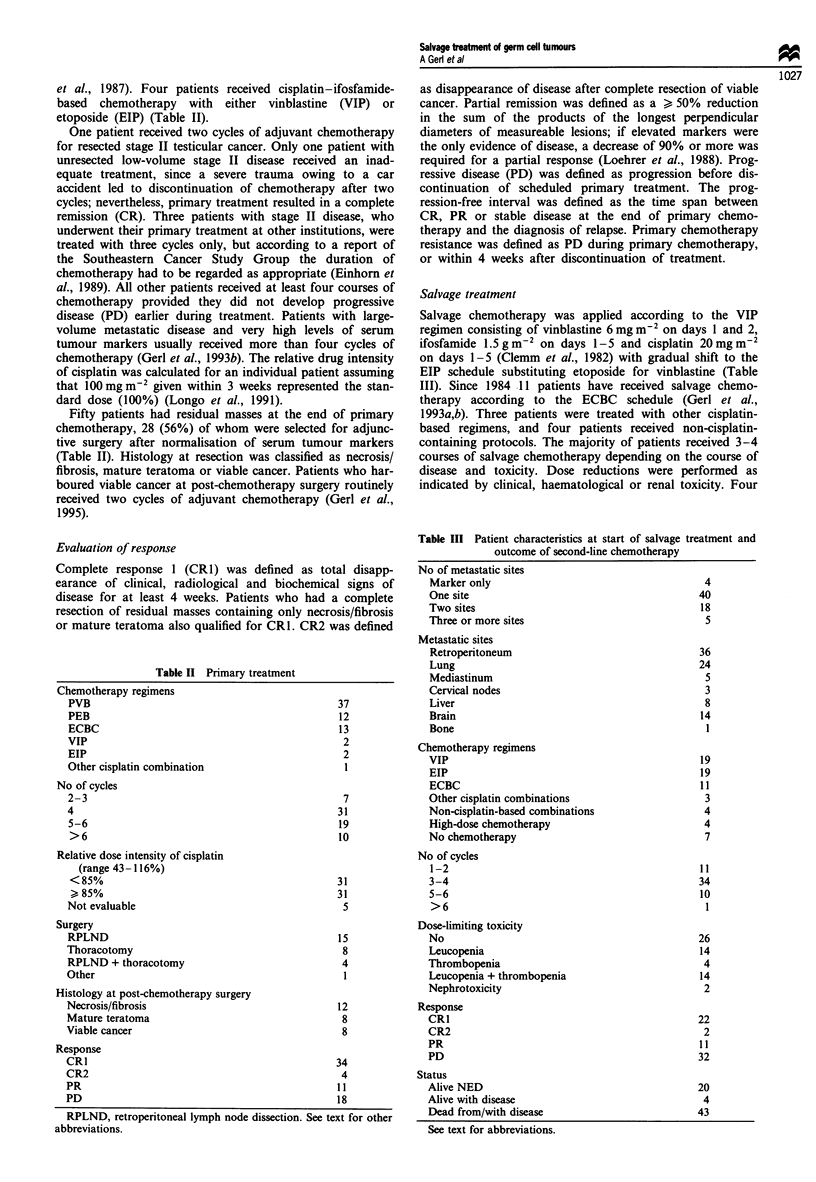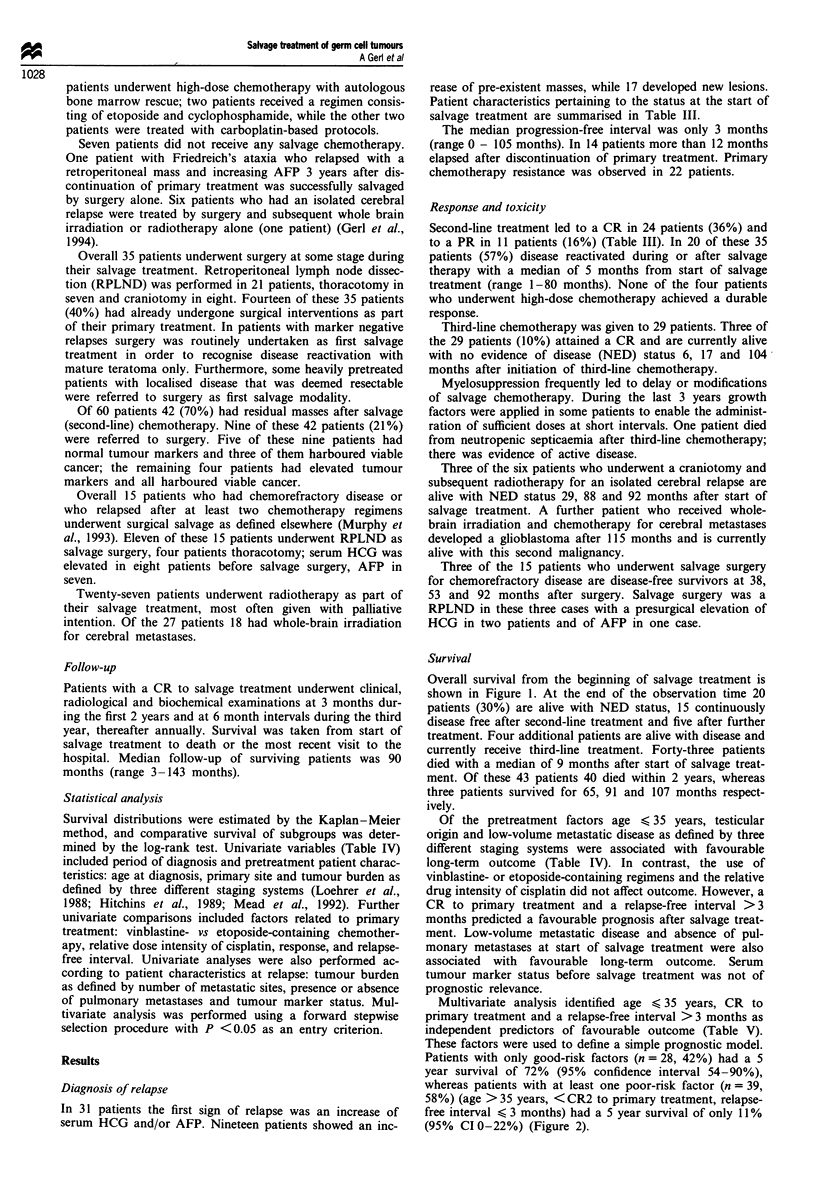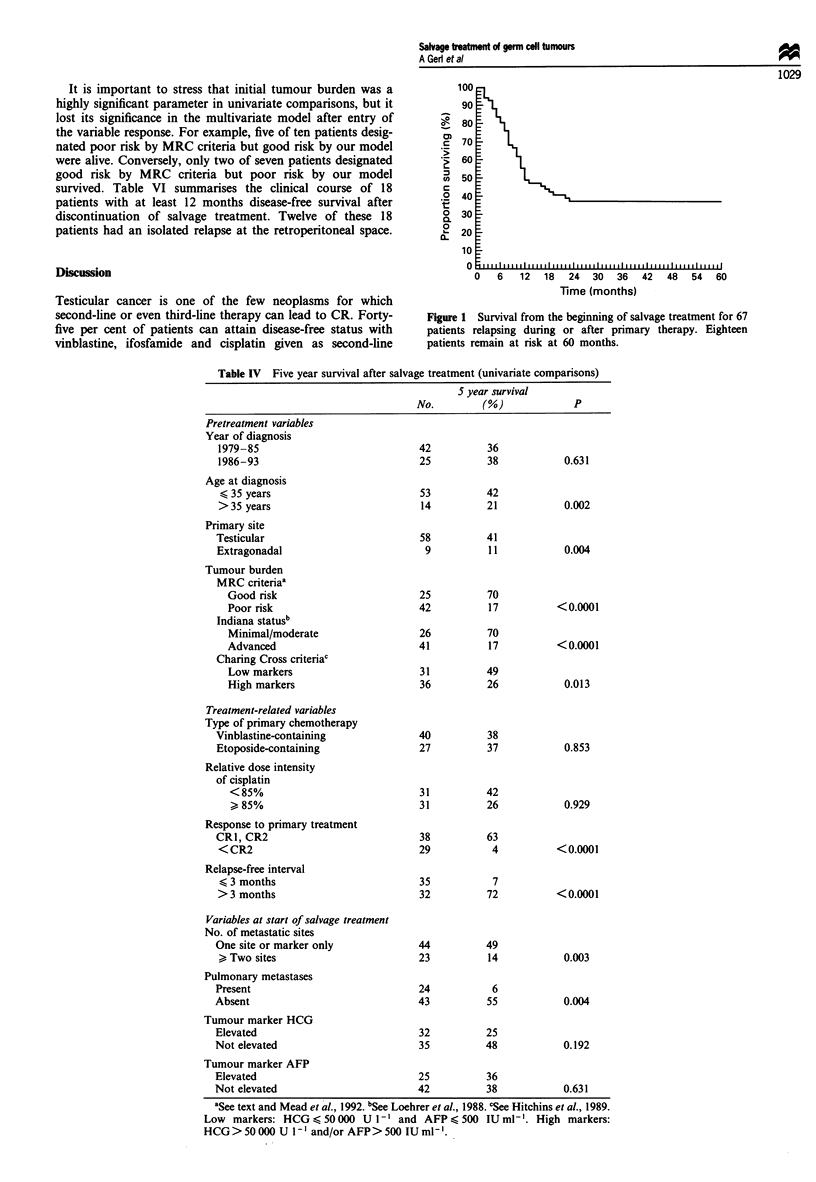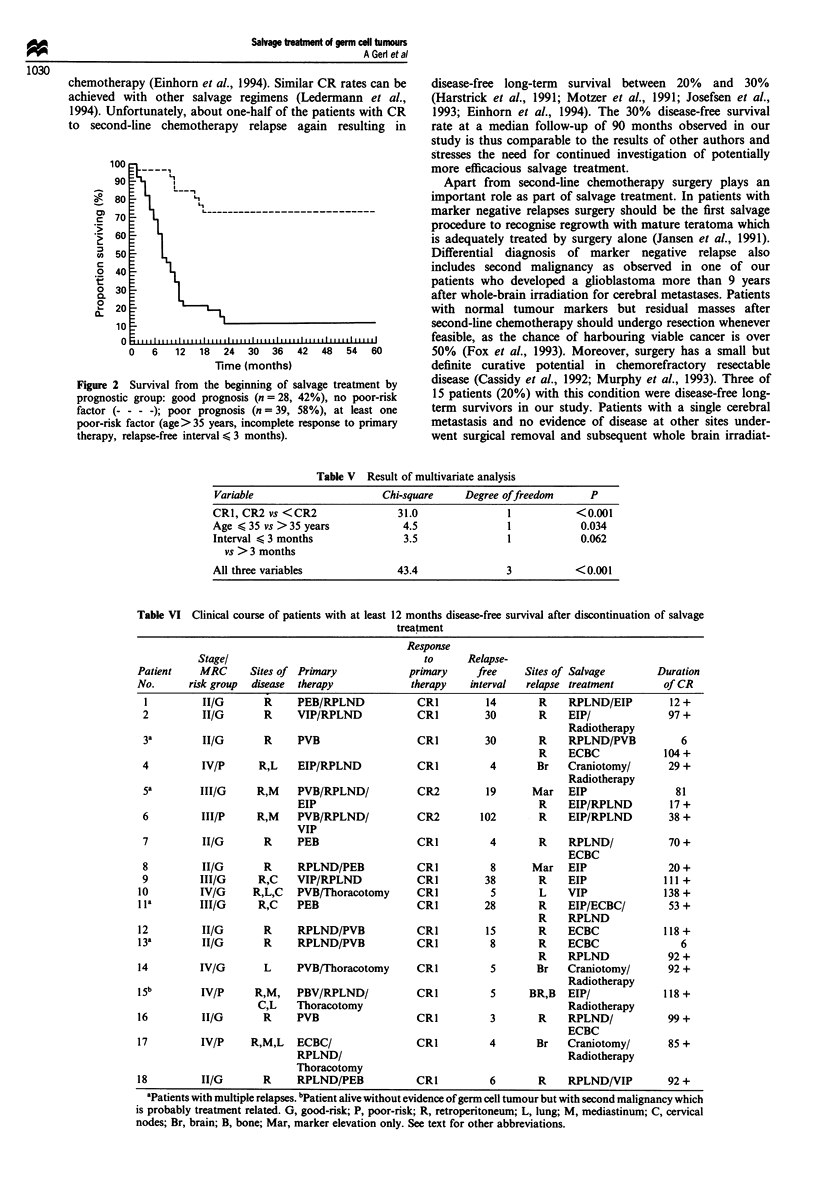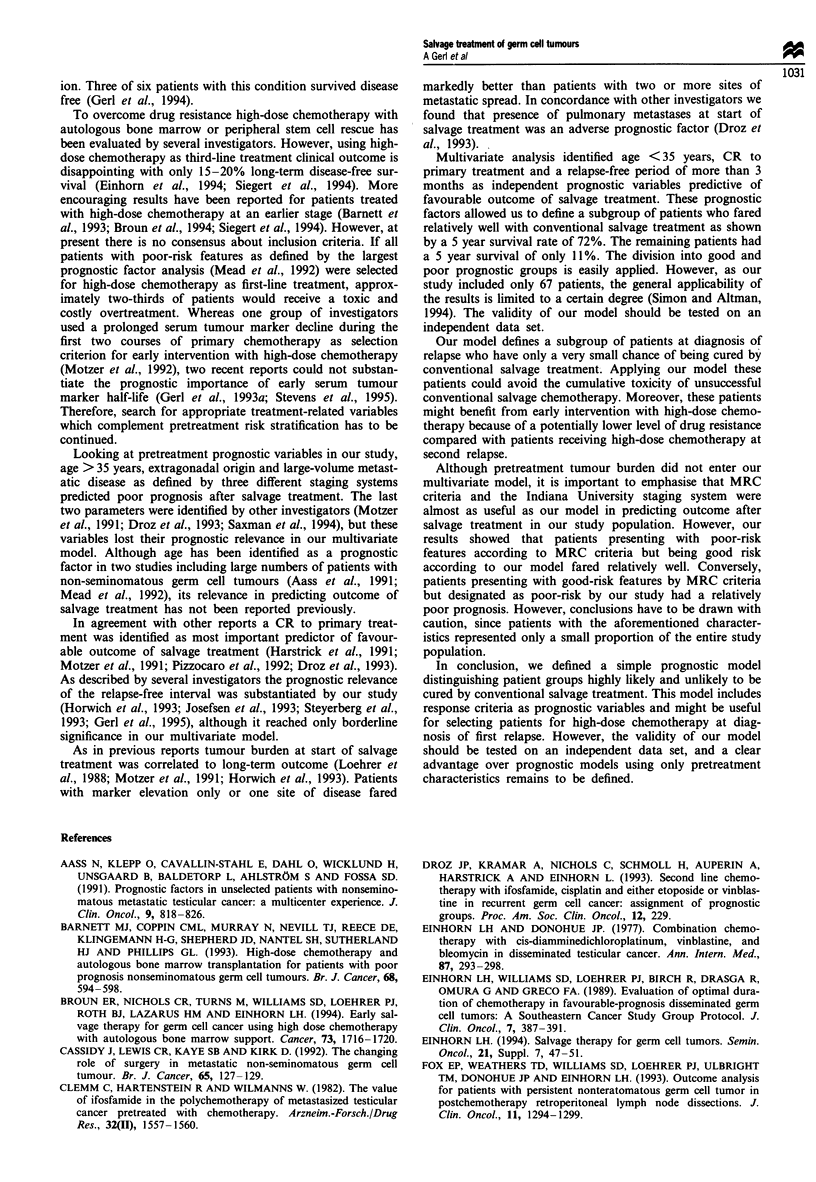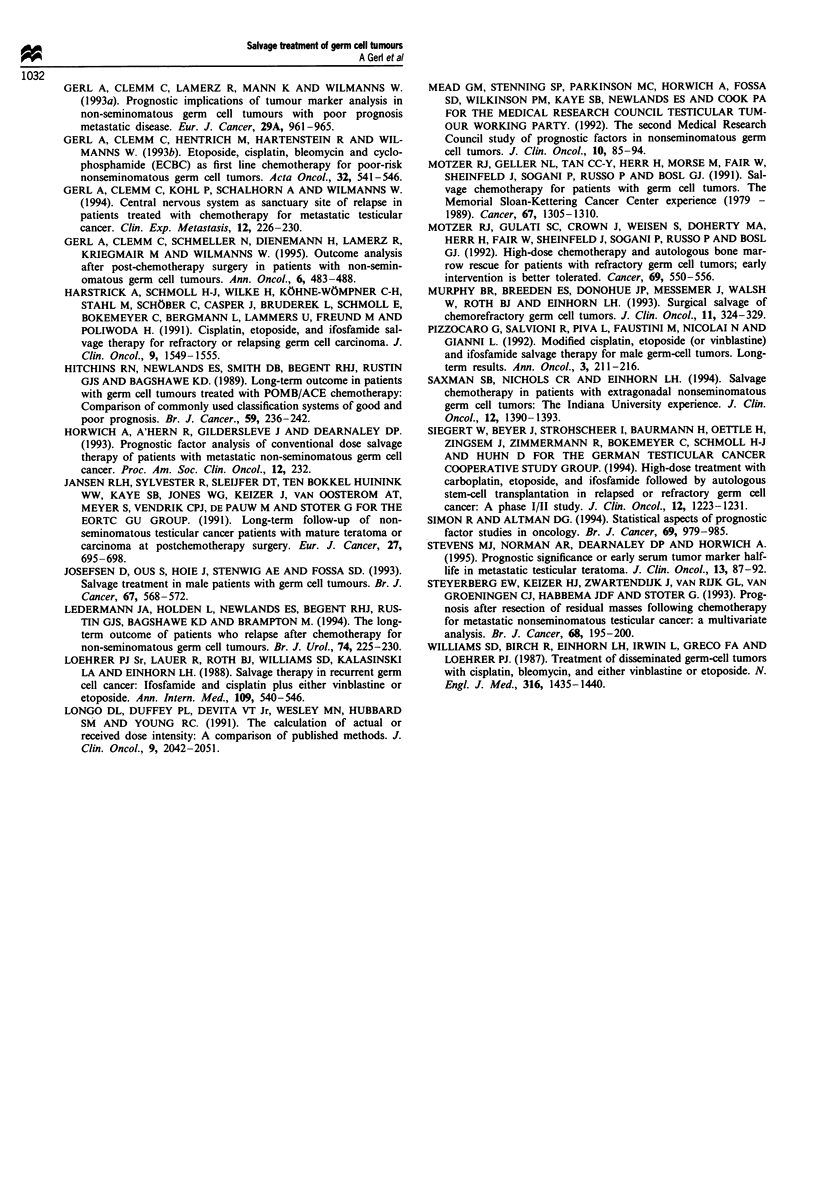# Prognosis after salvage treatment for unselected male patients with germ cell tumours.

**DOI:** 10.1038/bjc.1995.456

**Published:** 1995-10

**Authors:** A. Gerl, C. Clemm, N. Schmeller, R. Hartenstein, R. Lamerz, W. Wilmanns

**Affiliations:** Department of Internal Medicine III, Klinikum Grosshadern of the University of Munich, Germany.

## Abstract

Long-term outcome of salvage treatment was reviewed in 67 unselected male patients relapsing during or after their primary cisplatin-based chemotherapy for metastatic germ cell tumours. Seven patients underwent only surgery and/or radiotherapy as curatively intended salvage treatment. Thirty-five patients (52%) had a complete or partial response to salvage treatment, 20 (57%) of whom relapsed again. With a median follow-up of 90 months (range 3-143 months) 20 patients (30%) are alive with no evidence of disease, 15 continuously disease-free and five currently disease-free. The 5 year survival from start of salvage treatment is 37% for the group as a whole. Multivariate analysis identified age < or = 35 years, complete response to primary treatment and a relapse-free interval > 3 months as independent predictors of favourable outcome of salvage treatment. A group of patients with these good-risk factors (42%) had a 5 year survival of 72% compared with the remaining patients (58%) with a 5 year survival of only 11%. Whereas patients with good-risk features may be adequately managed by conventional salvage treatment, the remaining patients carry a very poor prognosis and require innovative and more aggressive approaches.


British Journal of Cancer (1995) 72, 1026–1032
© 1995 Stockton Press All rights reserved 0007-0920/95 $12.00

### Prognosis after salvage treatment for unselected male patients with germ cell tumours

A Gerl[1], C Clemm[2], N Schmeller[3], R Hartenstein[4], R Lamerz[5] and W Wilmanns[1,6]

[1]*Department of Internal Medicine III, Klinikum Grosshadern of the University of Munich;* [2]*Department of Internal Medicine, Clinic of Oncology Bad Trissl;* [3]*Department of Urology, Klinikum Grosshadern of the University of Munich;* [4]*Department of Internal Medicine IV, Munich Harlaching City Hospital;* [5]*Department of Internal Medicine II, Klinikum Grosshadern of the University of Munich;* [6]*GSF Forschungszentrum für Umwelt und Gesundheit, Munich, Germany.*

**Summary** Long-term outcome of salvage treatment was reviewed in 67 unselected male patients relapsing during or after their primary cisplatin-based chemotherapy for metastatic germ cell tumours. Seven patients underwent only surgery and/or radiotherapy as curatively intended salvage treatment. Thirty-five patients (52%) had a complete or partial response to salvage treatment, 20 (57%) of whom relapsed again. With a median follow-up of 90 months (range 3–143 months) 20 patients (30%) are alive with no evidence of disease, 15 continuously disease-free and five currently disease-free. The 5 year survival from start of salvage treatment is 37% for the group as a whole. Multivariate analysis identified age $\leqslant 35$ years, complete response to primary treatment and a relapse-free interval $> 3$ months as independent predictors of favourable outcome of salvage treatment. A group of patients with these good-risk factors (42%) had a 5 year survival of 72% compared with the remaining patients (58%) with a 5 year survival of only 11%. Whereas patients with good-risk features may be adequately managed by conventional salvage treatment, the remaining patients carry a very poor prognosis and require innovative and more aggressive approaches.

**Keywords:** chemotherapy; germ cell tumour; salvage treatment; testicular cancer

Cisplatin-based combination chemotherapy has considerably improved the outcome of patients with metastatic germ cell tumours. Around 75–80% of patients are cured by first-line treatment. However, 20–25% of patients develop disease progression during or after their initial chemotherapy and require effective salvage treatment. The prognosis of the latter patient group is relatively poor with only 20–30% disease-free long-term survival (Motzer *et al.*, 1991; Josefsen *et al.*, 1993; Einhorn *et al.*, 1994).

This retrospective study analyses outcome in patients who received salvage treatment for refractory or relapsing germ cell tumours at a single institution. We studied clinical features and response criteria as prognostic variables predictive of long-term survival after salvage treatment.

### Patients and methods

### *Patient characteristics*

Among 433 patients with germ cell tumours treated with cisplatin-based chemotherapy at Klinikum Grosshadern between 1979 and 1993, 61 developed signs of relapse during or after primary treatment. A further eleven patients, who received their primary treatment elsewhere, were referred to our institution for treatment of relapse. Relapse was defined as an increase of serum tumour markers human chorionic gonadotropin (HCG) and/or α-fetoprotein (AFP) requiring two samples, a greater than 25% increase of measurable lesions or development of a new lesion that proved to be viable cancer. Four patients who relapsed with mature teratoma or necrotic tumour tissue (one case) were thus excluded from this study. A further patient was excluded because of refusal of any salvage treatment. The remaining 67 patients were divided into two risk groups as defined by Medical Research Council criteria (Mead *et al.*, 1992). Patients with liver, bone, or brain metastases, a mediastinal mass $> 5$ cm, 20 or more lung metastases, AFP $> 1000$ IU $ml^{-1}$ and/or HCG $> 10\,000$ U $l^{-1}$ constituted a poor-risk group. Patients without any of these features were considered as good-risk. Patient characteristics pertaining to status immediately before initial chemotherapy are summarised in Table I.

### *Primary treatment*

Up to 1983 all patients received their primary chemotherapy according to the PVB protocol consisting of cisplatin 20 mg $m^{-2}$ on days 1–5, vinblastine 0.15–0.20 mg $kg^{-1}$ on days 1,2 and bleomycin 30 mg on days 2, 9, 16 (Einhorn and Donohue, 1977). Since 1984 patients with a large tumour burden have been predominantly treated according to the ECBC schedule consisting of etoposide 120 mg $m^{-2}$ on days 1–4, cisplatin 30 mg $m^{-2}$ on days 1–4, bleomycin 15 mg on day 1 (bolus) and 12 mg $m^{-2}$ on days 1–4 (24 h infusion), and cyclophosphamide 300 mg $m^{-2}$ on days 1–4 (Gerl *et al.*, 1993*a*,*b*). In 1987 we began to treat patients with low-volume metastatic disease according to the PEB regimen substituting etoposide 100 mg $m^{-2}$ on days 1–5 for vinblastine (Williams

**Table I** Patient characteristics

| | |
|---|---|
| No of patients | 67 |
| Age (years) | |
| Median | 27 |
| Range | 16–65 |
| Site | |
| Testicular | 58 |
| Extragonadal | 9 |
| Histology | |
| Seminoma | 2 |
| Non-seminoma | 65 |
| Stage | |
| II | 20 |
| III | 11 |
| IV | 36 |
| Risk groups (MRC criteria)[a] | |
| Good risk | 25 |
| Poor risk | 42 |

[a]See text.

Correspondence: A Gerl, Medizinische Klinik III, Klinikum Grosshadern, Marchioninistrasse 15, 81377 Munich, Germany
Received 3 February 1995; revised 26 April 1995; accepted 12 May 1995

et al., 1987). Four patients received cisplatin–ifosfamide-based chemotherapy with either vinblastine (VIP) or etoposide (EIP) (Table II).

One patient received two cycles of adjuvant chemotherapy for resected stage II testicular cancer. Only one patient with unresected low-volume stage II disease received an inadequate treatment, since a severe trauma owing to a car accident led to discontinuation of chemotherapy after two cycles; nevertheless, primary treatment resulted in a complete remission (CR). Three patients with stage II disease, who underwent their primary treatment at other institutions, were treated with three cycles only, but according to a report of the Southeastern Cancer Study Group the duration of chemotherapy had to be regarded as appropriate (Einhorn et al., 1989). All other patients received at least four courses of chemotherapy provided they did not develop progressive disease (PD) earlier during treatment. Patients with large-volume metastatic disease and very high levels of serum tumour markers usually received more than four cycles of chemotherapy (Gerl et al., 1993b). The relative drug intensity of cisplatin was calculated for an individual patient assuming that 100 mg $m^{-2}$ given within 3 weeks represented the standard dose (100%) (Longo et al., 1991).

Fifty patients had residual masses at the end of primary chemotherapy, 28 (56%) of whom were selected for adjunctive surgery after normalisation of serum tumour markers (Table II). Histology at resection was classified as necrosis/fibrosis, mature teratoma or viable cancer. Patients who harboured viable cancer at post-chemotherapy surgery routinely received two cycles of adjuvant chemotherapy (Gerl et al., 1995).

### Evaluation of response

Complete response 1 (CR1) was defined as total disappearance of clinical, radiological and biochemical signs of disease for at least 4 weeks. Patients who had a complete resection of residual masses containing only necrosis/fibrosis or mature teratoma also qualified for CR1. CR2 was defined as disappearance of disease after complete resection of viable cancer. Partial remission was defined as a ⩾50% reduction in the sum of the products of the longest perpendicular diameters of measureable lesions; if elevated markers were the only evidence of disease, a decrease of 90% or more was required for a partial response (Loehrer et al., 1988). Progressive disease (PD) was defined as progression before discontinuation of scheduled primary treatment. The progression-free interval was defined as the time span between CR, PR or stable disease at the end of primary chemotherapy and the diagnosis of relapse. Primary chemotherapy resistance was defined as PD during primary chemotherapy, or within 4 weeks after discontinuation of treatment.

### Salvage treatment

Salvage chemotherapy was applied according to the VIP regimen consisting of vinblastine 6 mg $m^{-2}$ on days 1 and 2, ifosfamide 1.5 g $m^{-2}$ on days 1–5 and cisplatin 20 mg $m^{-2}$ on days 1–5 (Clemm et al., 1982) with gradual shift to the EIP schedule substituting etoposide for vinblastine (Table III). Since 1984 11 patients have received salvage chemotherapy according to the ECBC schedule (Gerl et al., 1993a,b). Three patients were treated with other cisplatin-based regimens, and four patients received non-cisplatin-containing protocols. The majority of patients received 3–4 courses of salvage chemotherapy depending on the course of disease and toxicity. Dose reductions were performed as indicated by clinical, haematological or renal toxicity. Four

**Table II** Primary treatment

| | |
|---|---|
| Chemotherapy regimens | |
| PVB | 37 |
| PEB | 12 |
| ECBC | 13 |
| VIP | 2 |
| EIP | 2 |
| Other cisplatin combination | 1 |
| No of cycles | |
| 2–3 | 7 |
| 4 | 31 |
| 5–6 | 19 |
| >6 | 10 |
| Relative dose intensity of cisplatin (range 43–116%) | |
| <85% | 31 |
| ⩾85% | 31 |
| Not evaluable | 5 |
| Surgery | |
| RPLND | 15 |
| Thoracotomy | 8 |
| RPLND + thoracotomy | 4 |
| Other | 1 |
| Histology at post-chemotherapy surgery | |
| Necrosis/fibrosis | 12 |
| Mature teratoma | 8 |
| Viable cancer | 8 |
| Response | |
| CR1 | 34 |
| CR2 | 4 |
| PR | 11 |
| PD | 18 |

RPLND, retroperitoneal lymph node dissection. See text for other abbreviations.

**Table III** Patient characteristics at start of salvage treatment and outcome of second-line chemotherapy

| | |
|---|---|
| No of metastatic sites | |
| Marker only | 4 |
| One site | 40 |
| Two sites | 18 |
| Three or more sites | 5 |
| Metastatic sites | |
| Retroperitoneum | 36 |
| Lung | 24 |
| Mediastinum | 5 |
| Cervical nodes | 3 |
| Liver | 8 |
| Brain | 14 |
| Bone | 1 |
| Chemotherapy regimens | |
| VIP | 19 |
| EIP | 19 |
| ECBC | 11 |
| Other cisplatin combinations | 3 |
| Non-cisplatin-based combinations | 4 |
| High-dose chemotherapy | 4 |
| No chemotherapy | 7 |
| No of cycles | |
| 1–2 | 11 |
| 3–4 | 34 |
| 5–6 | 10 |
| >6 | 1 |
| Dose-limiting toxicity | |
| No | 26 |
| Leucopenia | 14 |
| Thrombopenia | 4 |
| Leucopenia + thrombopenia | 14 |
| Nephrotoxicity | 2 |
| Response | |
| CR1 | 22 |
| CR2 | 2 |
| PR | 11 |
| PD | 32 |
| Status | |
| Alive NED | 20 |
| Alive with disease | 4 |
| Dead from/with disease | 43 |

See text for abbreviations.

patients underwent high-dose chemotherapy with autologous bone marrow rescue; two patients received a regimen consisting of etoposide and cyclophosphamide, while the other two patients were treated with carboplatin-based protocols.

Seven patients did not receive any salvage chemotherapy. One patient with Friedreich's ataxia who relapsed with a retroperitoneal mass and increasing AFP 3 years after discontinuation of primary treatment was successfully salvaged by surgery alone. Six patients who had an isolated cerebral relapse were treated by surgery and subsequent whole brain irradiation or radiotherapy alone (one patient) (Gerl *et al.*, 1994).

Overall 35 patients underwent surgery at some stage during their salvage treatment. Retroperitoneal lymph node dissection (RPLND) was performed in 21 patients, thoracotomy in seven and craniotomy in eight. Fourteen of these 35 patients (40%) had already undergone surgical interventions as part of their primary treatment. In patients with marker negative relapses surgery was routinely undertaken as first salvage treatment in order to recognise disease reactivation with mature teratoma only. Furthermore, some heavily pretreated patients with localised disease that was deemed resectable were referred to surgery as first salvage modality.

Of 60 patients 42 (70%) had residual masses after salvage (second-line) chemotherapy. Nine of these 42 patients (21%) were referred to surgery. Five of these nine patients had normal tumour markers and three of them harboured viable cancer; the remaining four patients had elevated tumour markers and all harboured viable cancer.

Overall 15 patients who had chemorefractory disease or who relapsed after at least two chemotherapy regimens underwent surgical salvage as defined elsewhere (Murphy *et al.*, 1993). Eleven of these 15 patients underwent RPLND as salvage surgery, four patients thoracotomy; serum HCG was elevated in eight patients before salvage surgery, AFP in seven.

Twenty-seven patients underwent radiotherapy as part of their salvage treatment, most often given with palliative intention. Of the 27 patients 18 had whole-brain irradiation for cerebral metastases.

*Follow-up*

Patients with a CR to salvage treatment underwent clinical, radiological and biochemical examinations at 3 months during the first 2 years and at 6 month intervals during the third year, thereafter annually. Survival was taken from start of salvage treatment to death or the most recent visit to the hospital. Median follow-up of surviving patients was 90 months (range 3–143 months).

*Statistical analysis*

Survival distributions were estimated by the Kaplan–Meier method, and comparative survival of subgroups was determined by the log-rank test. Univariate variables (Table IV) included period of diagnosis and pretreatment patient characteristics: age at diagnosis, primary site and tumour burden as defined by three different staging systems (Loehrer *et al.*, 1988; Hitchins *et al.*, 1989; Mead *et al.*, 1992). Further univariate comparisons included factors related to primary treatment: vinblastine- *vs* etoposide-containing chemotherapy, relative dose intensity of cisplatin, response, and relapse-free interval. Univariate analyses were also performed according to patient characteristics at relapse: tumour burden as defined by number of metastatic sites, presence or absence of pulmonary metastases and tumour marker status. Multivariate analysis was performed using a forward stepwise selection procedure with $P < 0.05$ as an entry criterion.

### Results

*Diagnosis of relapse*

In 31 patients the first sign of relapse was an increase of serum HCG and/or AFP. Nineteen patients showed an increase of pre-existent masses, while 17 developed new lesions. Patient characteristics pertaining to the status at the start of salvage treatment are summarised in Table III.

The median progression-free interval was only 3 months (range 0 – 105 months). In 14 patients more than 12 months elapsed after discontinuation of primary treatment. Primary chemotherapy resistance was observed in 22 patients.

*Response and toxicity*

Second-line treatment led to a CR in 24 patients (36%) and to a PR in 11 patients (16%) (Table III). In 20 of these 35 patients (57%) disease reactivated during or after salvage therapy with a median of 5 months from start of salvage treatment (range 1–80 months). None of the four patients who underwent high-dose chemotherapy achieved a durable response.

Third-line chemotherapy was given to 29 patients. Three of the 29 patients (10%) attained a CR and are currently alive with no evidence of disease (NED) status 6, 17 and 104 months after initiation of third-line chemotherapy.

Myelosuppression frequently led to delay or modifications of salvage chemotherapy. During the last 3 years growth factors were applied in some patients to enable the administration of sufficient doses at short intervals. One patient died from neutropenic septicaemia after third-line chemotherapy; there was evidence of active disease.

Three of the six patients who underwent a craniotomy and subsequent radiotherapy for an isolated cerebral relapse are alive with NED status 29, 88 and 92 months after start of salvage treatment. A further patient who received whole-brain irradiation and chemotherapy for cerebral metastases developed a glioblastoma after 115 months and is currently alive with this second malignancy.

Three of the 15 patients who underwent salvage surgery for chemorefractory disease are disease-free survivors at 38, 53 and 92 months after surgery. Salvage surgery was a RPLND in these three cases with a presurgical elevation of HCG in two patients and of AFP in one case.

*Survival*

Overall survival from the beginning of salvage treatment is shown in Figure 1. At the end of the observation time 20 patients (30%) are alive with NED status, 15 continuously disease free after second-line treatment and five after further treatment. Four additional patients are alive with disease and currently receive third-line treatment. Forty-three patients died with a median of 9 months after start of salvage treatment. Of these 43 patients 40 died within 2 years, whereas three patients survived for 65, 91 and 107 months respectively.

Of the pretreatment factors age $\leqslant 35$ years, testicular origin and low-volume metastatic disease as defined by three different staging systems were associated with favourable long-term outcome (Table IV). In contrast, the use of vinblastine- or etoposide-containing regimens and the relative drug intensity of cisplatin did not affect outcome. However, a CR to primary treatment and a relapse-free interval $> 3$ months predicted a favourable prognosis after salvage treatment. Low-volume metastatic disease and absence of pulmonary metastases at start of salvage treatment were also associated with favourable long-term outcome. Serum tumour marker status before salvage treatment was not of prognostic relevance.

Multivariate analysis identified age $\leqslant 35$ years, CR to primary treatment and a relapse-free interval $> 3$ months as independent predictors of favourable outcome (Table V). These factors were used to define a simple prognostic model. Patients with only good-risk factors ($n = 28$, 42%) had a 5 year survival of 72% (95% confidence interval 54–90%), whereas patients with at least one poor-risk factor ($n = 39$, 58%) (age $> 35$ years, $<$CR2 to primary treatment, relapse-free interval $\leqslant 3$ months) had a 5 year survival of only 11% (95% CI 0–22%) (Figure 2).

It is important to stress that initial tumour burden was a highly significant parameter in univariate comparisons, but it lost its significance in the multivariate model after entry of the variable response. For example, five of ten patients designated poor risk by MRC criteria but good risk by our model were alive. Conversely, only two of seven patients designated good risk by MRC criteria but poor risk by our model survived. Table VI summarises the clinical course of 18 patients with at least 12 months disease-free survival after discontinuation of salvage treatment. Twelve of these 18 patients had an isolated relapse at the retroperitoneal space.

### Discussion

Testicular cancer is one of the few neoplasms for which second-line or even third-line therapy can lead to CR. Forty-five per cent of patients can attain disease-free status with vinblastine, ifosfamide and cisplatin given as second-line

**Figure 1** Survival from the beginning of salvage treatment for 67 patients relapsing during or after primary therapy. Eighteen patients remain at risk at 60 months.

**Table IV** Five year survival after salvage treatment (univariate comparisons)

| | No. | 5 year survival (%) | P |
|---|---|---|---|
| *Pretreatment variables* | | | |
| Year of diagnosis | | | |
| 1979–85 | 42 | 36 | |
| 1986–93 | 25 | 38 | 0.631 |
| Age at diagnosis | | | |
| ⩽35 years | 53 | 42 | |
| >35 years | 14 | 21 | 0.002 |
| Primary site | | | |
| Testicular | 58 | 41 | |
| Extragonadal | 9 | 11 | 0.004 |
| Tumour burden | | | |
| MRC criteria[a] | | | |
| Good risk | 25 | 70 | |
| Poor risk | 42 | 17 | <0.0001 |
| Indiana status[b] | | | |
| Minimal/moderate | 26 | 70 | |
| Advanced | 41 | 17 | <0.0001 |
| Charing Cross criteria[c] | | | |
| Low markers | 31 | 49 | |
| High markers | 36 | 26 | 0.013 |
| *Treatment-related variables* | | | |
| Type of primary chemotherapy | | | |
| Vinblastine-containing | 40 | 38 | |
| Etoposide-containing | 27 | 37 | 0.853 |
| Relative dose intensity of cisplatin | | | |
| <85% | 31 | 42 | |
| ⩾85% | 31 | 26 | 0.929 |
| Response to primary treatment | | | |
| CR1, CR2 | 38 | 63 | |
| <CR2 | 29 | 4 | <0.0001 |
| Relapse-free interval | | | |
| ⩽3 months | 35 | 7 | |
| >3 months | 32 | 72 | <0.0001 |
| *Variables at start of salvage treatment* | | | |
| No. of metastatic sites | | | |
| One site or marker only | 44 | 49 | |
| ⩾Two sites | 23 | 14 | 0.003 |
| Pulmonary metastases | | | |
| Present | 24 | 6 | |
| Absent | 43 | 55 | 0.004 |
| Tumour marker HCG | | | |
| Elevated | 32 | 25 | |
| Not elevated | 35 | 48 | 0.192 |
| Tumour marker AFP | | | |
| Elevated | 25 | 36 | |
| Not elevated | 42 | 38 | 0.631 |

[a]See text and Mead *et al.*, 1992. [b]See Loehrer *et al.*, 1988. [c]See Hitchins *et al.*, 1989. Low markers: HCG ⩽ 50 000 U $l^{-1}$ and AFP ⩽ 500 IU $ml^{-1}$. High markers: HCG > 50 000 U $l^{-1}$ and/or AFP > 500 IU $ml^{-1}$.

chemotherapy (Einhorn et al., 1994). Similar CR rates can be achieved with other salvage regimens (Ledermann et al., 1994). Unfortunately, about one-half of the patients with CR to second-line chemotherapy relapse again resulting in disease-free long-term survival between 20% and 30% (Harstrick et al., 1991; Motzer et al., 1991; Josefsen et al., 1993; Einhorn et al., 1994). The 30% disease-free survival rate at a median follow-up of 90 months observed in our study is thus comparable to the results of other authors and stresses the need for continued investigation of potentially more efficacious salvage treatment.

**Figure 2** Survival from the beginning of salvage treatment by prognostic group: good prognosis ($n = 28$, 42%), no poor-risk factor (- - - -); poor prognosis ($n = 39$, 58%), at least one poor-risk factor (age > 35 years, incomplete response to primary therapy, relapse-free interval ⩽ 3 months).

Apart from second-line chemotherapy surgery plays an important role as part of salvage treatment. In patients with marker negative relapses surgery should be the first salvage procedure to recognise regrowth with mature teratoma which is adequately treated by surgery alone (Jansen et al., 1991). Differential diagnosis of marker negative relapse also includes second malignancy as observed in one of our patients who developed a glioblastoma more than 9 years after whole-brain irradiation for cerebral metastases. Patients with normal tumour markers but residual masses after second-line chemotherapy should undergo resection whenever feasible, as the chance of harbouring viable cancer is over 50% (Fox et al., 1993). Moreover, surgery has a small but definite curative potential in chemorefractory resectable disease (Cassidy et al., 1992; Murphy et al., 1993). Three of 15 patients (20%) with this condition were disease-free long-term survivors in our study. Patients with a single cerebral metastasis and no evidence of disease at other sites underwent surgical removal and subsequent whole brain irradiat-

**Table V** Result of multivariate analysis

| *Variable* | *Chi-square* | *Degree of freedom* | *P* |
|---|---|---|---|
| CR1, CR2 vs <CR2 | 31.0 | 1 | <0.001 |
| Age ⩽ 35 vs >35 years | 4.5 | 1 | 0.034 |
| Interval ⩽ 3 months vs >3 months | 3.5 | 1 | 0.062 |
| All three variables | 43.4 | 3 | <0.001 |

**Table VI** Clinical course of patients with at least 12 months disease-free survival after discontinuation of salvage treatment

| *Patient No.* | *Stage/ MRC risk group* | *Sites of disease* | *Primary therapy* | *Response to primary therapy* | *Relapse-free interval* | *Sites of relapse* | *Salvage treatment* | *Duration of CR* |
|---|---|---|---|---|---|---|---|---|
| 1 | II/G | R | PEB/RPLND | CR1 | 14 | R | RPLND/EIP | 12+ |
| 2 | II/G | R | VIP/RPLND | CR1 | 30 | R | EIP/ Radiotherapy | 97+ |
| 3[a] | II/G | R | PVB | CR1 | 30 | R<br>R | RPLND/PVB<br>ECBC | 6<br>104+ |
| 4 | IV/P | R,L | EIP/RPLND | CR1 | 4 | Br | Craniotomy/ Radiotherapy | 29+ |
| 5[a] | III/G | R,M | PVB/RPLND/ EIP | CR2 | 19 | Mar<br>R | EIP<br>EIP/RPLND | 81<br>17+ |
| 6 | III/P | R,M | PVB/RPLND/ VIP | CR2 | 102 | R | EIP/RPLND | 38+ |
| 7 | II/G | R | PEB | CR1 | 4 | R | RPLND/ ECBC | 70+ |
| 8 | II/G | R | RPLND/PEB | CR1 | 8 | Mar | EIP | 20+ |
| 9 | III/G | R,C | VIP/RPLND | CR1 | 38 | R | EIP | 111+ |
| 10 | IV/G | R,L,C | PVB/Thoracotomy | CR1 | 5 | L | VIP | 138+ |
| 11[a] | III/G | R,C | PEB | CR1 | 28 | R<br>R | EIP/ECBC/<br>RPLND | 53+ |
| 12 | II/G | R | RPLND/PVB | CR1 | 15 | R | ECBC | 118+ |
| 13[a] | II/G | R | RPLND/PVB | CR1 | 8 | R<br>R | ECBC<br>RPLND | 6<br>92+ |
| 14 | IV/G | L | PVB/Thoracotomy | CR1 | 5 | Br | Craniotomy/ Radiotherapy | 92+ |
| 15[b] | IV/P | R,M, C,L | PBV/RPLND/ Thoracotomy | CR1 | 5 | BR,B | EIP/ Radiotherapy | 118+ |
| 16 | II/G | R | PVB | CR1 | 3 | R | RPLND/ ECBC | 99+ |
| 17 | IV/P | R,M,L | ECBC/ RPLND/ Thoracotomy | CR1 | 4 | Br | Craniotomy/ Radiotherapy | 85+ |
| 18 | II/G | R | RPLND/PEB | CR1 | 6 | R | RPLND/VIP | 92+ |

[a]Patients with multiple relapses. [b]Patient alive without evidence of germ cell tumour but with second malignancy which is probably treatment related. G, good-risk; P, poor-risk; R, retroperitoneum; L, lung; M, mediastinum; C, cervical nodes; Br, brain; B, bone; Mar, marker elevation only. See text for other abbreviations.

ion. Three of six patients with this condition survived disease free (Gerl *et al.*, 1994).

To overcome drug resistance high-dose chemotherapy with autologous bone marrow or peripheral stem cell rescue has been evaluated by several investigators. However, using high-dose chemotherapy as third-line treatment clinical outcome is disappointing with only 15–20% long-term disease-free survival (Einhorn *et al.*, 1994; Siegert *et al.*, 1994). More encouraging results have been reported for patients treated with high-dose chemotherapy at an earlier stage (Barnett *et al.*, 1993; Broun *et al.*, 1994; Siegert *et al.*, 1994). However, at present there is no consensus about inclusion criteria. If all patients with poor-risk features as defined by the largest prognostic factor analysis (Mead *et al.*, 1992) were selected for high-dose chemotherapy as first-line treatment, approximately two-thirds of patients would receive a toxic and costly overtreatment. Whereas one group of investigators used a prolonged serum tumour marker decline during the first two courses of primary chemotherapy as selection criterion for early intervention with high-dose chemotherapy (Motzer *et al.*, 1992), two recent reports could not substantiate the prognostic importance of early serum tumour marker half-life (Gerl *et al.*, 1993*a*; Stevens *et al.*, 1995). Therefore, search for appropriate treatment-related variables which complement pretreatment risk stratification has to be continued.

Looking at pretreatment prognostic variables in our study, age >35 years, extragonadal origin and large-volume metastatic disease as defined by three different staging systems predicted poor prognosis after salvage treatment. The last two parameters were identified by other investigators (Motzer *et al.*, 1991; Droz *et al.*, 1993; Saxman *et al.*, 1994), but these variables lost their prognostic relevance in our multivariate model. Although age has been identified as a prognostic factor in two studies including large numbers of patients with non-seminomatous germ cell tumours (Aass *et al.*, 1991; Mead *et al.*, 1992), its relevance in predicting outcome of salvage treatment has not been reported previously.

In agreement with other reports a CR to primary treatment was identified as most important predictor of favourable outcome of salvage treatment (Harstrick *et al.*, 1991; Motzer *et al.*, 1991; Pizzocaro *et al.*, 1992; Droz *et al.*, 1993). As described by several investigators the prognostic relevance of the relapse-free interval was substantiated by our study (Horwich *et al.*, 1993; Josefsen *et al.*, 1993; Steyerberg *et al.*, 1993; Gerl *et al.*, 1995), although it reached only borderline significance in our multivariate model.

As in previous reports tumour burden at start of salvage treatment was correlated to long-term outcome (Loehrer *et al.*, 1988; Motzer *et al.*, 1991; Horwich *et al.*, 1993). Patients with marker elevation only or one site of disease fared markedly better than patients with two or more sites of metastatic spread. In concordance with other investigators we found that presence of pulmonary metastases at start of salvage treatment was an adverse prognostic factor (Droz *et al.*, 1993).

Multivariate analysis identified age <35 years, CR to primary treatment and a relapse-free period of more than 3 months as independent prognostic variables predictive of favourable outcome of salvage treatment. These prognostic factors allowed us to define a subgroup of patients who fared relatively well with conventional salvage treatment as shown by a 5 year survival rate of 72%. The remaining patients had a 5 year survival of only 11%. The division into good and poor prognostic groups is easily applied. However, as our study included only 67 patients, the general applicability of the results is limited to a certain degree (Simon and Altman, 1994). The validity of our model should be tested on an independent data set.

Our model defines a subgroup of patients at diagnosis of relapse who have only a very small chance of being cured by conventional salvage treatment. Applying our model these patients could avoid the cumulative toxicity of unsuccessful conventional salvage chemotherapy. Moreover, these patients might benefit from early intervention with high-dose chemotherapy because of a potentially lower level of drug resistance compared with patients receiving high-dose chemotherapy at second relapse.

Although pretreatment tumour burden did not enter our multivariate model, it is important to emphasise that MRC criteria and the Indiana University staging system were almost as useful as our model in predicting outcome after salvage treatment in our study population. However, our results showed that patients presenting with poor-risk features according to MRC criteria but being good risk according to our model fared relatively well. Conversely, patients presenting with good-risk features by MRC criteria but designated as poor-risk by our study had a relatively poor prognosis. However, conclusions have to be drawn with caution, since patients with the aforementioned characteristics represented only a small proportion of the entire study population.

In conclusion, we defined a simple prognostic model distinguishing patient groups highly likely and unlikely to be cured by conventional salvage treatment. This model includes response criteria as prognostic variables and might be useful for selecting patients for high-dose chemotherapy at diagnosis of first relapse. However, the validity of our model should be tested on an independent data set, and a clear advantage over prognostic models using only pretreatment characteristics remains to be defined.

### References

AASS N, KLEPP O, CAVALLIN-STAHL E, DAHL O, WICKLUND H, UNSGAARD B, BALDETORP L, AHLSTRÖM S AND FOSSA SD. (1991). Prognostic factors in unselected patients with nonseminomatous metastatic testicular cancer: a multicenter experience. *J. Clin. Oncol.*, **9**, 818–826.

BARNETT MJ, COPPIN CML, MURRAY N, NEVILL TJ, REECE DE, KLINGEMANN H-G, SHEPHERD JD, NANTEL SH, SUTHERLAND HJ AND PHILLIPS GL. (1993). High-dose chemotherapy and autologous bone marrow transplantation for patients with poor prognosis nonseminomatous germ cell tumours. *Br. J. Cancer*, **68**, 594–598.

BROUN ER, NICHOLS CR, TURNS M, WILLIAMS SD, LOEHRER PJ, ROTH BJ, LAZARUS HM AND EINHORN LH. (1994). Early salvage therapy for germ cell cancer using high dose chemotherapy with autologous bone marrow support. *Cancer*, **73**, 1716–1720.

CASSIDY J, LEWIS CR, KAYE SB AND KIRK D. (1992). The changing role of surgery in metastatic non-seminomatous germ cell tumour. *Br. J. Cancer*, **65**, 127–129.

CLEMM C, HARTENSTEIN R AND WILMANNS W. (1982). The value of ifosfamide in the polychemotherapy of metastasized testicular cancer pretreated with chemotherapy. *Arzneim.-Forsch./Drug Res.*, **32(II)**, 1557–1560.

DROZ JP, KRAMAR A, NICHOLS C, SCHMOLL H, AUPERIN A, HARSTRICK A AND EINHORN L. (1993). Second line chemotherapy with ifosfamide, cisplatin and either etoposide or vinblastine in recurrent germ cell cancer: assignment of prognostic groups. *Proc. Am. Soc. Clin. Oncol.*, **12**, 229.

EINHORN LH AND DONOHUE JP. (1977). Combination chemotherapy with cis-diamminedichloroplatinum, vinblastine, and bleomycin in disseminated testicular cancer. *Ann. Intern. Med.*, **87**, 293–298.

EINHORN LH, WILLIAMS SD, LOEHRER PJ, BIRCH R, DRASGA R, OMURA G AND GRECO FA. (1989). Evaluation of optimal duration of chemotherapy in favourable-prognosis disseminated germ cell tumors: A Southeastern Cancer Study Group Protocol. *J. Clin. Oncol.*, **7**, 387–391.

EINHORN LH. (1994). Salvage therapy for germ cell tumors. *Semin. Oncol.*, **21**, Suppl. 7, 47–51.

FOX EP, WEATHERS TD, WILLIAMS SD, LOEHRER PJ, ULBRIGHT TM, DONOHUE JP AND EINHORN LH. (1993). Outcome analysis for patients with persistent nonteratomatous germ cell tumor in postchemotherapy retroperitoneal lymph node dissections. *J. Clin. Oncol.*, **11**, 1294–1299.

GERL A, CLEMM C, LAMERZ R, MANN K AND WILMANNS W. (1993*a*). Prognostic implications of tumour marker analysis in non-seminomatous germ cell tumours with poor prognosis metastatic disease. *Eur. J. Cancer*, **29A**, 961–965.

GERL A, CLEMM C, HENTRICH M, HARTENSTEIN R AND WILMANNS W. (1993*b*). Etoposide, cisplatin, bleomycin and cyclophosphamide (ECBC) as first line chemotherapy for poor-risk nonseminomatous germ cell tumors. *Acta Oncol.*, **32**, 541–546.

GERL A, CLEMM C, KOHL P, SCHALHORN A AND WILMANNS W. (1994). Central nervous system as sanctuary site of relapse in patients treated with chemotherapy for metastatic testicular cancer. *Clin. Exp. Metastasis*, **12**, 226–230.

GERL A, CLEMM C, SCHMELLER N, DIENEMANN H, LAMERZ R, KRIEGMAIR M AND WILMANNS W. (1995). Outcome analysis after post-chemotherapy surgery in patients with non-seminomatous germ cell tumours. *Ann. Oncol.*, **6**, 483–488.

HARSTRICK A, SCHMOLL H-J, WILKE H, KÖHNE-WÖMPNER C-H, STAHL M, SCHÖBER C, CASPER J, BRUDEREK L, SCHMOLL E, BOKEMEYER C, BERGMANN L, LAMMERS U, FREUND M AND POLIWODA H. (1991). Cisplatin, etoposide, and ifosfamide salvage therapy for refractory or relapsing germ cell carcinoma. *J. Clin. Oncol.*, **9**, 1549–1555.

HITCHINS RN, NEWLANDS ES, SMITH DB, BEGENT RHJ, RUSTIN GJS AND BAGSHAWE KD. (1989). Long-term outcome in patients with germ cell tumours treated with POMB/ACE chemotherapy: Comparison of commonly used classification systems of good and poor prognosis. *Br. J. Cancer.*, **59**, 236–242.

HORWICH A, A'HERN R, GILDERSLEVE J AND DEARNALEY DP. (1993). Prognostic factor analysis of conventional dose salvage therapy of patients with metastatic non-seminomatous germ cell cancer. *Proc. Am. Soc. Clin. Oncol.*, **12**, 232.

JANSEN RLH, SYLVESTER R, SLEIJFER DT, TEN BOKKEL HUININK WW, KAYE SB, JONES WG, KEIZER J, VAN OOSTEROM AT, MEYER S, VENDRIK CPJ, DE PAUW M AND STOTER G FOR THE EORTC GU GROUP. (1991). Long-term follow-up of non-seminomatous testicular cancer patients with mature teratoma or carcinoma at postchemotherapy surgery. *Eur. J. Cancer*, **27**, 695–698.

JOSEFSEN D, OUS S, HOIE J, STENWIG AE AND FOSSA SD. (1993). Salvage treatment in male patients with germ cell tumours. *Br. J. Cancer*, **67**, 568–572.

LEDERMANN JA, HOLDEN L, NEWLANDS ES, BEGENT RHJ, RUSTIN GJS, BAGSHAWE KD AND BRAMPTON M. (1994). The long-term outcome of patients who relapse after chemotherapy for non-seminomatous germ cell tumours. *Br. J. Urol.*, **74**, 225–230.

LOEHRER PJ Sr, LAUER R, ROTH BJ, WILLIAMS SD, KALASINSKI LA AND EINHORN LH. (1988). Salvage therapy in recurrent germ cell cancer: Ifosfamide and cisplatin plus either vinblastine or etoposide. *Ann. Intern. Med.*, **109**, 540–546.

LONGO DL, DUFFEY PL, DEVITA VT Jr, WESLEY MN, HUBBARD SM AND YOUNG RC. (1991). The calculation of actual or received dose intensity: A comparison of published methods. *J. Clin. Oncol.*, **9**, 2042–2051.

MEAD GM, STENNING SP, PARKINSON MC, HORWICH A, FOSSA SD, WILKINSON PM, KAYE SB, NEWLANDS ES AND COOK PA FOR THE MEDICAL RESEARCH COUNCIL TESTICULAR TUMOUR WORKING PARTY. (1992). The second Medical Research Council study of prognostic factors in nonseminomatous germ cell tumors. *J. Clin. Oncol.*, **10**, 85–94.

MOTZER RJ, GELLER NL, TAN CC-Y, HERR H, MORSE M, FAIR W, SHEINFELD J, SOGANI P, RUSSO P AND BOSL GJ. (1991). Salvage chemotherapy for patients with germ cell tumors. The Memorial Sloan-Kettering Cancer Center experience (1979 – 1989). *Cancer*, **67**, 1305–1310.

MOTZER RJ, GULATI SC, CROWN J, WEISEN S, DOHERTY MA, HERR H, FAIR W, SHEINFELD J, SOGANI P, RUSSO P AND BOSL GJ. (1992). High-dose chemotherapy and autologous bone marrow rescue for patients with refractory germ cell tumors; early intervention is better tolerated. *Cancer*, **69**, 550–556.

MURPHY BR, BREEDEN ES, DONOHUE JP, MESSEMER J, WALSH W, ROTH BJ AND EINHORN LH. (1993). Surgical salvage of chemorefractory germ cell tumors. *J. Clin. Oncol.*, **11**, 324–329.

PIZZOCARO G, SALVIONI R, PIVA L, FAUSTINI M, NICOLAI N AND GIANNI L. (1992). Modified cisplatin, etoposide (or vinblastine) and ifosfamide salvage therapy for male germ-cell tumors. Long-term results. *Ann. Oncol.*, **3**, 211–216.

SAXMAN SB, NICHOLS CR AND EINHORN LH. (1994). Salvage chemotherapy in patients with extragonadal nonseminomatous germ cell tumors: The Indiana University experience. *J. Clin. Oncol.*, **12**, 1390–1393.

SIEGERT W, BEYER J, STROHSCHEER I, BAURMANN H, OETTLE H, ZINGSEM J, ZIMMERMANN R, BOKEMEYER C, SCHMOLL H-J AND HUHN D FOR THE GERMAN TESTICULAR CANCER COOPERATIVE STUDY GROUP. (1994). High-dose treatment with carboplatin, etoposide, and ifosfamide followed by autologous stem-cell transplantation in relapsed or refractory germ cell cancer: A phase I/II study. *J. Clin. Oncol.*, **12**, 1223–1231.

SIMON R AND ALTMAN DG. (1994). Statistical aspects of prognostic factor studies in oncology. *Br. J. Cancer*, **69**, 979–985.

STEVENS MJ, NORMAN AR, DEARNALEY DP AND HORWICH A. (1995). Prognostic significance or early serum tumor marker half-life in metastatic testicular teratoma. *J. Clin. Oncol.*, **13**, 87–92.

STEYERBERG EW, KEIZER HJ, ZWARTENDIJK J, VAN RIJK GL, VAN GROENINGEN CJ, HABBEMA JDF AND STOTER G. (1993). Prognosis after resection of residual masses following chemotherapy for metastatic nonseminomatous testicular cancer: a multivariate analysis. *Br. J. Cancer*, **68**, 195–200.

WILLIAMS SD, BIRCH R, EINHORN LH, IRWIN L, GRECO FA AND LOEHRER PJ. (1987). Treatment of disseminated germ-cell tumors with cisplatin, bleomycin, and either vinblastine or etoposide. *N. Engl. J. Med.*, **316**, 1435–1440.